# Physical interactions between DNA and sepiolite nanofibers, and potential application for DNA transfer into mammalian cells

**DOI:** 10.1038/srep36341

**Published:** 2016-11-03

**Authors:** Fidel Antonio Castro-Smirnov, Olivier Piétrement, Pilar Aranda, Jean-Rémi Bertrand, Jeanne Ayache, Eric Le Cam, Eduardo Ruiz-Hitzky, Bernard S. Lopez

**Affiliations:** 1 CNRS UMR 8200, Institut de Cancérologie Gustave-Roussy, Université Paris-Saclay, team labeled “Ligue 2014”, 114 rue Edouard Vaillant, 94805 Villejuif, France; 2 Universidad de las Ciencias Informáticas, Carretera a San Antonio de los Baños, km 2 1⁄2, La Habana 19370, Cuba; 3 CNRS UMR 8126, Gustave Roussy, Université Paris-Saclay, F-94805 Villejuif, France; 4 Instituto de Ciencia de Materiales de Madrid (ICMM-CSIC), c/Sor Juana Inés de la Cruz 3, 28049 Madrid, Spain; 5 Vectorology and Anticancer therapies, UMR 8203, CNRS, Université Paris-Saclay, Gustave Roussy, Université Paris-Saclay, 94805 Villejuif, France

## Abstract

Nanofibers of sepiolite, a natural silicate belonging to the clay minerals family, might constitute a potential promising nanocarrier for the non-viral transfer of bio-molecules. We show here that sepiolite nanofibers efficiently bind different types of DNA molecules through electrostatic interactions, hydrogen bonding, cation bridges, and van der Waals forces. Moreover, Fourier-transform infrared spectroscopy identified the external silanol groups as the main sites of interaction with the DNA. Furthermore, as a proof of concept, we show that sepiolite is able to stably transfer plasmid DNA into mammalian cells and that the efficiency can be optimized. Indeed, sonication of sepiolite 100-fold stimulated DNA transfection efficiency. These results open the way to the use of sepiolite-based biohybrids as a novel class of nanoplatform for gene transfer with potential clinical applications.

The transfer of DNA into biological organisms is a pivotal issue for the design of novel and promising strategies for gene therapy and/or the development of new biological models of interest for both academic and applied medical, biotechnological and agronomic research^1^. Remarkably, the development of novel nanocarriers using biohybrid materials, such as bionanocomposites, for non-viral gene transfer constitutes a promising approach for the treatment of genetic disorders, cardiovascular diseases, AIDS, Alzheimer’s disease, multiple sclerosis and cancer^2^,^3^,^4^,^5^,^6^.

Clay minerals (natural silicates of aluminum and/or magnesium) represent one of the most abundant groups of inorganic solids that interact with the biosphere^7^. These minerals have been implicated in the prebiotic synthesis of biomolecules at the origin of life^8^. Because of their functional properties, bionanohybrid and bionanocomposite materials resulting from the combination of biopolymers with nanoparticulate solids such as clay minerals^9^,^10^,^11^,^12^ represent alluring prospects for biomedical applications^13^,^14^,^15^,^16^, such as biosensors, scaffolds for tissue engineering, effective drug-delivery nanovehicles, vaccination, and wound dressings^17^,^18^,^19^,^20^,^21^,^22^,^23^,^24^,^25^,^26^,^27^,^28^.

Sepiolite is a natural hydrated magnesium silicate of microfibrous morphology with the theoretical unit cell formula of Si_12_O_30_Mg_8_(OH,F)_4_(H_2_O)_4_·8H_2_O^23^,^24^ provided with the ability to assemble a wide variety of organic and inorganic species^21^,^22^. Sepiolite exhibits an alternating structure of blocks and tunnels that grow up in the fiber direction (supplementary data S1). The blocks consist of two layers of tetrahedral silica sandwiching a central magnesium oxide-hydroxide layer. The discontinuity of the silica sheets leads to the presence of silanol groups (Si-OH) at the edges of the channels, representing tunnels that are open to the external surface of the sepiolite particles^25^. The tunnels and channels are filled with two types of water molecules, including a) coordinated water molecules, which are bound to the Mg^2+^ ions located at the edges of the octahedral sheets, and b) zeolitic water, which is associated with the former ions through hydrogen bonding. One of the advantages of fibrous clays, such as sepiolite, compared to “conventional” clays based on layered silicates (i.e., smectites and vermiculites) is the high density of surface silanol groups, which allows hydrogen bonding interactions at the silicate interacting with diverse organic species^21^. Moreover, this silicate possesses a cation-exchange capacity (10–20 mEq/100 g), which is the result of partial isomorphous substitutions of magnesium by aluminum and other trivalent metals in the octahedral sheet of the structure of sepiolite. Therefore, the negatively charged surface may also ensure ionic interactions in the assembly of sepiolite with inorganic cations, in addition to positively charged molecular species.

The unique properties of the sepiolite surface (large specific surface area, negative electric charge, and fibrous morphology) make this clay mineral a potential platform for the co-delivery of different types of bioactive species. Indeed, sepiolite has been shown to interact with polysaccharides^12^,^26^,^27^, lipids^28^, proteins^29^,^30^,^31^ and virus particles^18^,^32^, leading to a significant diversity of bionanocomposites^13^. Importantly, the length of the nanofibers in the sepiolite selected in this work makes it suitable for potential cellular uptake. Additionally, sepiolite possesses stable intrinsic fluorescence properties. Therefore, by taking advantage of this natural fluorescence, it should be possible to select cells containing sepiolite using conventional cell sorting techniques. Finally, sepiolite is a natural and abundant silicate that is considered non-hazardous and non-carcinogenic by the International Agency of Research on Cancer (IARC)^33^. Consistently, *in vitro* and *in vivo* tests, as well as epidemiological studies, have also confirmed that sepiolite from Taxus Basin deposits in Spain does not constitute a health risk^34^.

DNA transfer into bacteria has been described through a process named Yoshida effect^35^. This process involves the formation of holes resulting from the friction forces when spreading bacteria on agar plates, in the bacteria membrane that then allows the uptake of the DNA. However, the Yoshida effect cannot work with mammalian cells because cells are not spread on agar plate, and, in addition, friction forces would kill the cells. Therefore, in mammalian cells, the strategy should either use the natural and spontaneous internalization into cells through biological processes such as endocytosis or macropinocytosis or transfer using chemical or physical treatment of the cells. However, in both cases, the use of the sepiolite as a carrier implies that the complex DNA/sepiolite should be first assemble prior to add it to the cells.

Here, we first focus on the synthesis and physicochemical characterization of new DNA-based bionanocomposites, in which nucleic acids are adsorbed onto sepiolite nanofibrous clay. We report that sepiolite efficiently binds DNA, introducing a deeply detailed study on the interaction mechanisms between sepiolite and DNA, as well as physicochemical characterizations of the resulting sepiolite/DNA (Sep/DNA) bionanocomposites. Second, as a proof of concept, we show the potential capacity of sepiolite to stably deliver DNA into mammalian cells.

## Results and Discussion

### Synthesis of Sep/DNA bionanocomposites (Sep/DNA)

**Figure 1:**
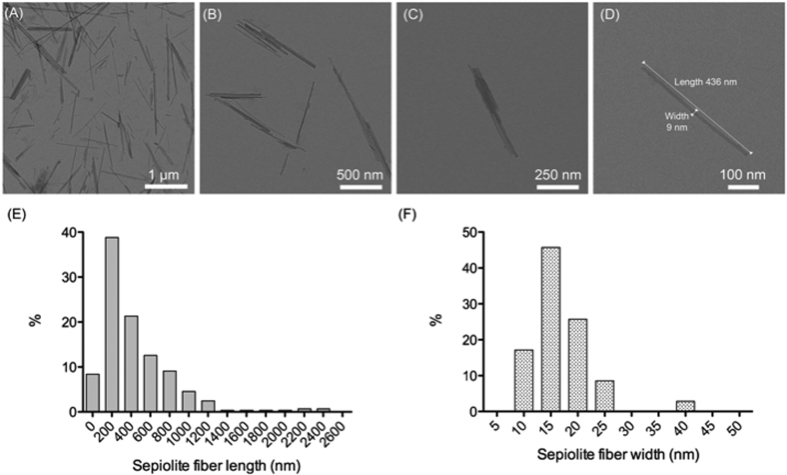
Size distribution of sepiolite fibers. (**A–D**) TEM images of sepiolite fibers (1 mg/ml sepiolite dispersion in 10 mM Tris-HCl, pH = 7.5) (**E**) sepiolite fiber length distribution (nm), (**F**) sepiolite fiber width distribution (nm).

In this work we used a commercial sepiolite obtained from the Vallecas-Vicalvaro deposits near Madrid, Spain (see Experimental Section). First, the sepiolite fibers (present in water dispersions) were analyzed by transmission electron microscopy (TEM) to determine the fiber size distribution. The mean value for the fiber width was 15 nm, and approximately 80% of fibers were between 200 and 400 nm long, with a maximal length of 800 nm (Fig. 1). Therefore, their small size and the reported low toxicity^34^ of sepiolite nanofibers makes this clay material potentially suitable for intracellular access into mammalian cells.

**Figure 2:**
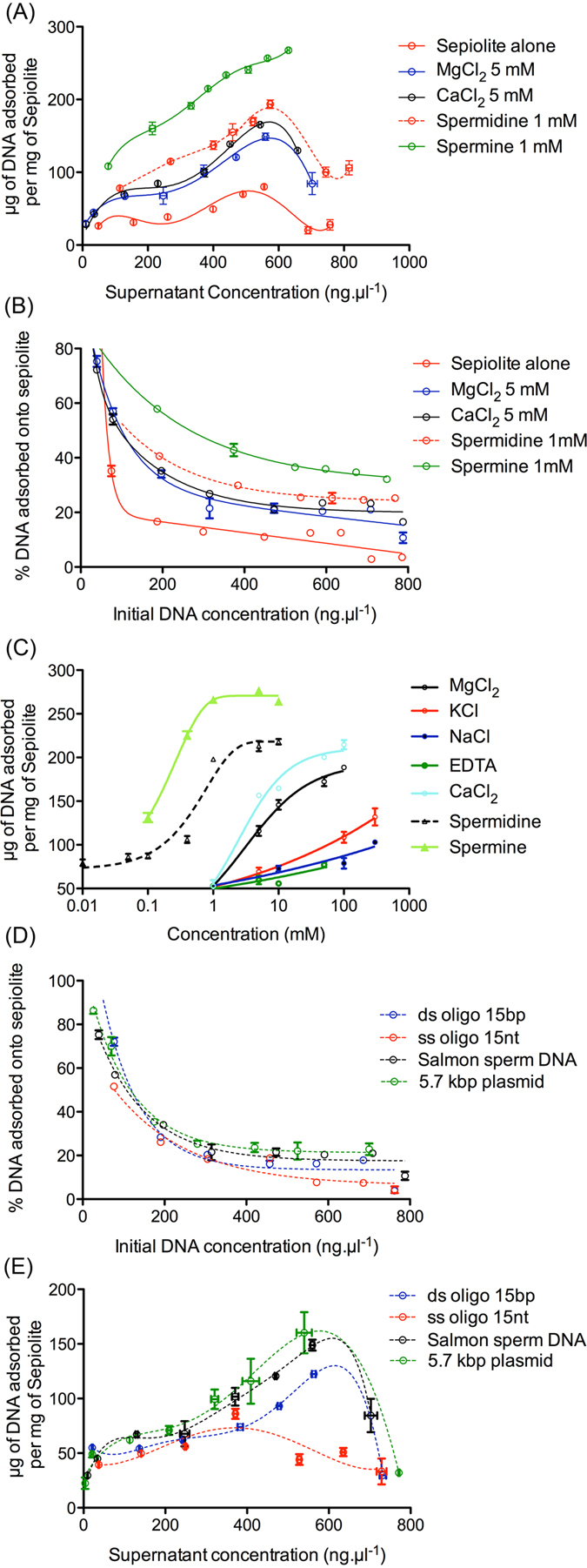
(**A**,**B**) Adsorption isotherms of DNA on sepiolite in the presence of various polyvalent cations. Each point has error bars for 3 different experiments. Reaction conditions: 10 mM Tris-HCl pH = 7.5 and a sepiolite concentration of 1 mg/ml. (**C**) The effect of the presence of cations with different valences on DNA adsorption. Reaction conditions: 10 mM Tris-HCl pH = 7.5, salmon sperm DNA and sepiolite concentrations of 615 ng·μl^−1^ and 1 mg/ml, respectively. (**D**,**E**) Adsorption isotherms of different DNA conformations on sepiolite. Reaction conditions: 10 mM Tris-HCl pH = 7.5, 5 mM MgCl_2_, sepiolite concentration of 1 mg/ml; 50 μg of sepiolite was used in each experiment. Adsorption occurred at 25 °C for 24 hours under agitation at 700 rpm using an Eppendorf Thermomixer.

Adsorption isotherms revealed the efficient adsorption of DNA onto sepiolite nanofibers from Tris-HCl buffer solution at pH 7.5 (Fig. 2A). At low initial concentrations of DNA and concentrations up to 400 ng·μl^−1^, the adsorption behavior follows a convex simple-component Langmuir isotherm. At higher DNA concentrations (>400 ng·μl^−1^), the curves indicate a multi-layer adsorption mechanism and saturation. The saturation point was found for an initial DNA concentration of 630 ng·μl^−1^, corresponding to the maximum of spontaneous DNA adsorption onto sepiolite (approximately 80 μg of DNA adsorbed per mg of sepiolite). At the highest concentrations of added DNA (>630 ng·μl^−1^), the adsorption abruptly decreased. This evidence shows that the adsorption process depends on both the initial DNA and sepiolite concentrations. For DNA initial concentrations lower than 100 ng·μl^−1^, more than 30% of the DNA was adsorbed. This value dropped to 12% for initial DNA concentrations ranging from 200 ng·μl^−1^ to 650 ng·μl^−1^ and was less than 10% for initial DNA concentrations higher than 650 ng·μl^−1^ (Fig. 2B).

Supplying multivalent cations, such as either magnesium or calcium or spermidine or spermine, to the suspension significantly increased the adsorption of DNA onto sepiolite (Fig. 2A,B). This observation suggests that the presence of any of these cations favors the adsorption process, most likely as a result of cation bridge interactions^36^,^37^ together with electrostatic interactions between DNA and sepiolite surface. Indeed, divalent cations may interact with the Si-OH groups of the sepiolite surface, reducing electrostatic repulsion between the DNA and the sepiolite and acting as bridges for binding DNA molecules, as it has been reported for DNA interactions between DNA and other types of silicate substrates^38^. Moreover, cation binding may reduce intramolecular electrostatic repulsion among the DNA molecule subunits, leading to higher DNA diffusion coefficients due to the more compact conformation of the molecule^38^.

More specifically, divalent cations lead to a two-fold increase in DNA adsorption (Fig. 2A). In this case, the maximum adsorption was found in samples prepared at an initial DNA concentration of 700 ng·μl^−1^, which led to 160 μg of DNA adsorbed per mg of sepiolite. DNA bound slightly more efficiently to sepiolite in the presence of CaCl_2_ compared to MgCl_2_. This could be attributed to the fact that Mg^2+^ forms weaker “outer-sphere” complexes with the phosphate backbone, while Ca^2+^ forms “inner-sphere” complexes with the phosphate backbone of plasmid DNA, which decreases the net negative charge and hence decreases the electrostatic repulsion between phosphates and results in a more compact plasmid molecular structure and a higher diffusion coefficient^38^.

Polyamine cations, such as spermidine (trivalent) and spermine (tetravalent) cations, stimulated more efficient adsorption than divalent cations, as previously shown for the mica surface^39^. In the presence of spermidine, the isotherm curves retained the same shape with a maximum in the adsorption corresponding to the same initial DNA concentration relative to that of the experiments carried out in the presence of divalent cations, although approximately 200 μg of adsorbed DNA was achieved per mg of sepiolite. With this system, it is possible to achieve more than 100 μg of adsorbed DNA, even when the initial DNA concentration is higher than 750 ng·μl^−1^ (Fig. 2A). In the presence of spermine, adsorption was significantly more efficient, and no decrease in DNA adsorption was observed for initial concentrations of DNA higher than 750 ng·μl^−1^, in contrast to experiments carried out in presence of divalent and trivalent cations. Contrasting with the other systems, the maximum obtained value for adsorption was found for initial DNA concentrations as high as 750 ng·μl^−1^, corresponding to 270 μg of DNA adsorbed per mg of sepiolite. These results can be explained by the DNA-condensing ability of polyamines, which lead to sharp increases in local DNA concentration in proximity of the sepiolite surface, where these ions should be located.

Moreover, from the isotherm curves, it is clear that the presence of multivalent cations also increased the percentage of initial DNA adsorbed onto sepiolite, given the same initial DNA concentrations (Fig. 2B). In the presence of divalent and trivalent cations, 20% to 30% of DNA was adsorbed at initial concentrations ranging from 300 to 700 ng·μl^−1^, increasing to more than 50% when the initial concentration was lower than 100 ng·μl^−1^. In the presence of the tetravalent cation, from 30% to 40% of DNA was adsorbed at initial concentrations higher than 400 ng·μl^−1^; the percentage increased to more than 50% at initial concentrations less than 300 ng·μl^−1^ (Fig. 2B).

The overall adsorption process appears to occur very rapidly. Indeed, for 50 μg of sepiolite, we observed only a slight increase (approximately 3%) in the total amount of DNA adsorbed between a few minutes and 24 hours of DNA incubation with sepiolite in the presence of a divalent cation (MgCl_2_) (Supplementary data S2). This result indicates that the majority of DNA molecules are instantaneously adsorbed onto the sepiolite, pointing out to the predominance of electrostatic interactions between sepiolite and DNA, in addition to hydrogen bonding with the silanol groups located on the external surface of the silicate.

To further examine the effect of cation valence on the adsorption of DNA onto sepiolite, we analyzed the impact of increasing concentrations of the studied cations in the medium, increasing them while keeping constant the DNA concentration (Fig. 2C). The efficiency of DNA adsorption appears to be directly correlated to the cation valence. Indeed, the tetravalent cation (spermine) was more efficient than the trivalent cation (spermidine), and both were significantly more efficient than the divalent cations (MgCl_2_ and CaCl_2_). For example, DNA adsorption was as efficient with 1 mM spermidine (trivalent cation) as with 100 mM divalent cation (MgCl_2_ and CaCl_2_). Spermine (tetravalent cation) proved to be even more efficient (Fig. 2C).

Surprisingly, the presence of monovalent cations (*e.g.*, NaCl and KCl) slightly favored DNA adsorption (Fig. 2C), which is not the case for other types of clay minerals. Monovalent cations cause the screening of electrostatic interactions on montmorillonite^38^ and decrease the correlation force involved in DNA adsorption on mica^40^. At a pH of 7.5, both DNA and the sepiolite surface are negatively charged; therefore, the presence of monovalent cations could screen the interaction between the silanol groups and the negative charges on the DNA phosphate backbone^41^, thus decreasing the adsorption of DNA. However, as previously proposed^39^, this charge screening effect could also reduce the electrostatic energy barrier between DNA and sepiolite, which would increase DNA adsorption by allowing the DNA molecules to approach the negative surface sufficiently close for adsorption via van der Waals forces.

Among the tested monovalent cations, KCl was slightly more efficient than NaCl. However, the use of 100 mM of KCl and 300 mM of NaCl lead to a DNA adsorption efficiency that was the same as that observed for 5 mM MgCl_2_. Additionally, the monovalent cations were three orders of magnitude less efficient than the tetravalent cation. Surprisingly, with EDTA chelating agent, a poor but significant residual efficiency of DNA adsorption was preserved (Fig. 2C).

In additional experiments, we compared the adsorption efficiency of different DNA conformations, *i.e.* sonicated linear double-stranded DNA (a mean of 300 bp), containing complex DNA sequences (from salmon sperm), circular plasmids (5.7 kb long), double-strand linear oligonucleotides, ds-oligo (15 bp) or linear single-strand oligonucleotides, ss-oligo (15 nt) (Fig. 2D,E). At initial DNA concentrations less than 300 ng·μl^−1^ and using 50 μg of sepiolite, the behaviors of all types of the studied DNA were quite similar. At initial DNA concentrations less than 100 ng·μl^−1^, the percentage of adsorbed DNA (from the initial sample) was greater than 50% (Fig. 2D), reaching adsorption values higher than 70% at initial concentrations less than 50 ng·μl^−1^ for double-stranded DNA. However, at higher initial concentrations, the adsorption isotherms exhibited significant differences between the adsorption of double-stranded and single-stranded DNA onto sepiolite (Fig. 2E). The double-stranded DNA presented maximum adsorption behavior at an initial concentration of approximately 700 ng·μl^−1^, which corresponds to an equilibrium concentration of approximately 550 ng·μl^−1^, after adsorption. Beyond this initial concentration value, the amount of adsorbed DNA decreases. With ss-oligo DNA, the maximum of adsorption was two-fold lower than that of double-stranded DNA (Fig. 2D), corresponding to an initial DNA concentration close to 460 ng·μl^−1^, which corresponds to an equilibrium concentration of 370 ng·μl^−1^.

To confirm the modifications of sepiolite surface charged sites, in the presence of multivalent cations and DNA molecules, the zeta-potential was determined in an electrokinetic study in the presence of different salts with and without DNA (Table 1).

The zeta-potential analysis of sepiolite with different salts in the medium confirmed that the presence of multivalent cations decreased the negative charge of the sepiolite and that the trivalent and tetravalent cations (spermidine and spermine, respectively) were the most efficient ions at this shielding effect, leading to more complete surface charge inversion. Nevertheless, the presence of DNA, which is able to interact with the external surface of the sepiolite fibers, leads to the formation of bionanocomposites with a net negative charge, either prepared with or without the presence of polyvalent cations. This result confirms that the interaction between DNA and the sepiolite surface implicates diverse mechanisms and indicates a complex assembly process.

**Figure 3:**
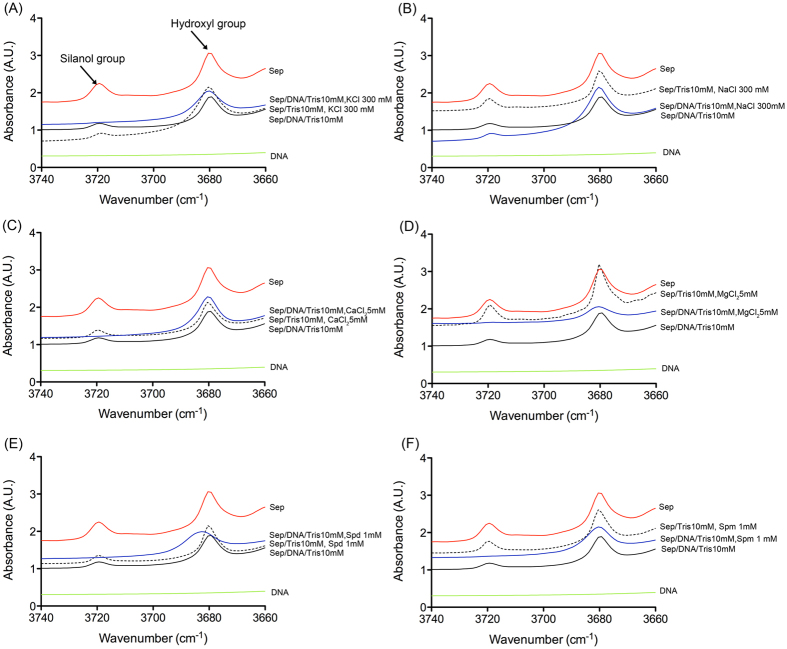
FTIR spectra of sepiolite and the Sep/DNA bionanocomposite prepared in 10 mM Tris-HCl pH = 7.5 compared to Sep/DNA bionanocomposites prepared in the presence of monovalent (**A**: NaCl, **B**: KCl), divalent (**C**: CaCl_2_, **D**: MgCl_2_), trivalent (**E**: spermidine) and tetravalent (**F**: spermine) cations.

To further elucidate the nature of the interactions between DNA and the sepiolite surface, Sep/DNA bionanocomposites were characterized by Fourier-transform infrared spectroscopy (FTIR). Indeed, this technique has been previously employed to ascertain the interaction of diverse biomolecules such as polysaccharides^27^, proteins^29^ and phospholipids^28^ with silanol groups at the external surface of sepiolite. Here, FTIR analysis revealed that the DNA adsorption specifically affected the intensity of the 3720 cm^−1^ band, assigned to the stretching O-H vibrations of Si-OH located on the external surface of sepiolite (Fig. 3) 21,25,27,42.

Indeed, the interaction of adsorbed DNA with these groups perturbed the OH stretching vibration band, producing a shift toward lower frequency values, precluding its observability in the spectrum due to overlap with the broad band associated with the stretching OH modes of water molecules belonging to the sepiolite^21^,^41^,^42^. Changes in the frequency of this band have been correlated with interactions of diverse type of species with the silanol group^17^,^21^,^26^,^27^,^28^,^32^. In contrast, the band assigned to the stretching OH vibration of Mg-OH at ca. 3680 cm^−1^ 21,25 remained unaltered even at high amounts of adsorbed DNA. Of note, this last type of hydroxyl group is located inside the structural blocks of sepiolite and is therefore inaccessible to DNA. Taken together, these data show that DNA binds to sepiolite through external silanol groups but not through the hydroxyl groups located inside the sepiolite tunnels. In the present study, the degree of the perturbation of this specific band (Fig. 3) might be correlated with the formation of a bionanocomposite in the presence of multivalent cations and thus with the efficiency of DNA adsorption onto sepiolite (compare Figs 2 and 3). A decrease in the intensity of the silanol band was observed to a different extent for each bionanocomposite, which may be correlated with variation in the surface coverage degree. In contrast, the band assigned to the stretching OH vibration of Mg-OH at ca. 3680 cm^−1^ remained almost unaltered even at high amounts of adsorbed DNA because this last type of hydroxyl group is located inside the structural blocks of sepiolite being inaccessible to DNA adsorbed molecules. As dimensions of the cross-section of the tunnels are approximately 1.1 × 0.4 nm^2^, these cavities are inaccessible to DNA, consistently with the bigger size of DNA. Note that a similar behavior has already been observed in other polymer/sepiolite nanocomposites^17^,^26^,^27^,^28^,^32^.

**Figure 4:**
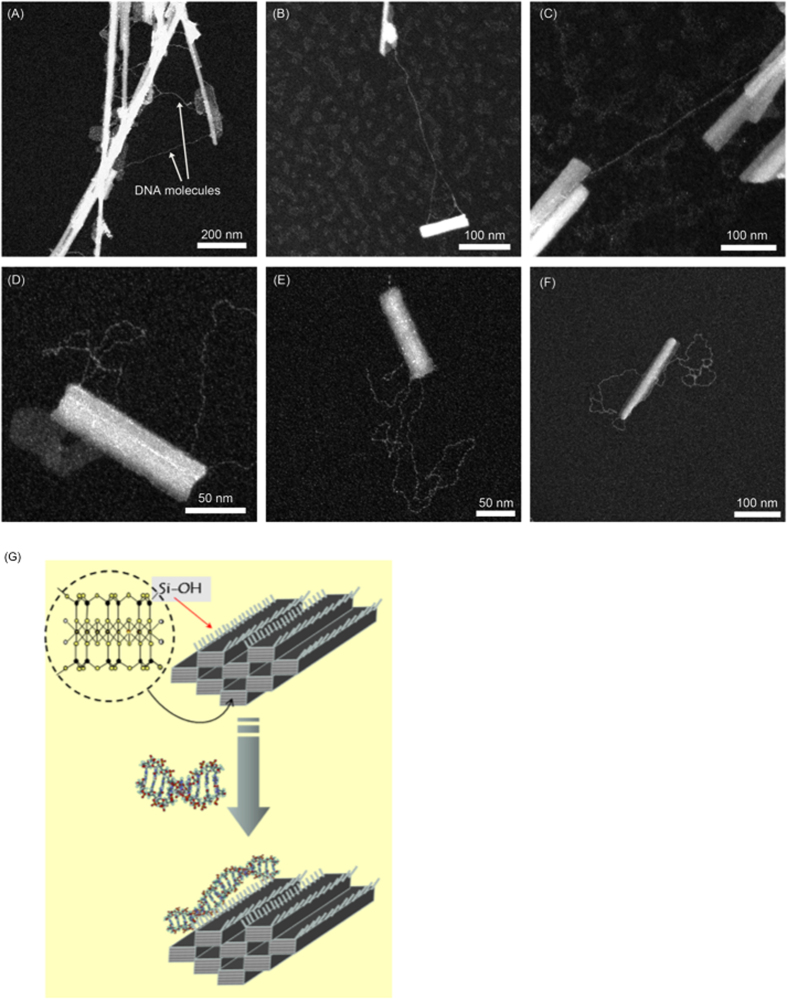
TEM characterization of the Sep/DNA bionanocomposite (**A–C**). (**D,E**) TEM images of a single molecule of Sep/DNA. The DNA bound to the sepiolite at the edge of the nanofiber and on its external surface. Nanofibers can be completely coated with DNA, but some DNA molecules are bound at one of their extremities, and two different nanofibers can be linked by a DNA plasmid. Reaction conditions: 1 mg/ml sepiolite, 10 mM Tris-HCl, pH = 7.5, 5 mM MgCl_2_, and 50 ng·μl^−1^ plasmid DNA (5.7 kb). (**G**) Scheme illustrating the DNA-sepiolite bionanocomposite resulting from the sepiolite interaction with linear DNA. It is also show the silanol groups (Si-OH) present at the external surface of the sepiolite fibers which are in hydrogen bonding interaction with nitrogenated bases at the DNA chains.

The TEM (Fig. 4A–F) and atomic force microscopy (AFM) (supplementary data S3) observations of the Sep/DNA bionanocomposites confirm that the DNA-sepiolite fiber assembly partially covers the surface of the silicate nanoparticles (Fig. 4G).

Consistent with the FTIR study, the information obtained from the AFM and TEM techniques shows that the DNA chains preferentially bonded to the sepiolite along the edge of the nanofibers and to diverse adsorption sites located on the external surface of the sepiolite. The nanofibers were not completely coated with DNA, and some DNA molecules were able to bind at either one or two fiber edges. Additionally, it is possible for more than two nanofibers to be linked by one DNA plasmid chain (Fig. 4).

### DNA desorption from sepiolite

**Figure 5:**
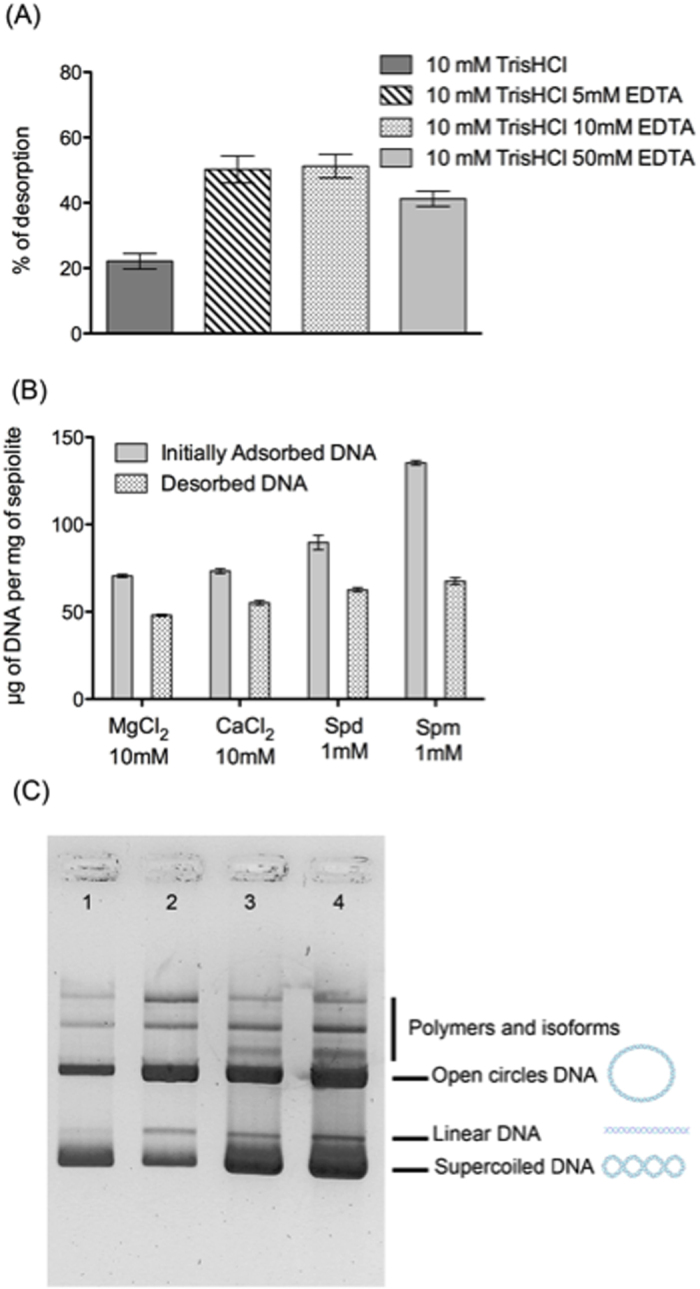
(**A**) Comparison of DNA desorption efficiency at different concentrations of EDTA. Reaction conditions: Initially, 0.5 ml of Sep/DNA samples were prepared using DNA at 640 ng·μl^−1^ in a sepiolite dispersion (1 mg/ml) in 10 mM Tris-HCl pH = 7.5 and 5 mM MgCl_2_. Resuspension in 0.1 ml solution of 10 mM Tris-HCl pH = 7.5, and EDTA at 5, 10 and 50 mM. After 15 min of incubation at room temperature, all samples were centrifuged at 5000 rpm for 5 min, and the supernatant was measured using the UV-vis. (**B**) Comparison of DNA desorption efficiency for Sep/DNA prepared in the presence of Mg^2+^, Ca^2+^, spermidine (Spd) or spermine (Spm). (**C**) Characterization with EMSA of desorbed DNA using the “chelation” method. 1) plasmid (5.7 kbp) control; 2) plasmid DNA in the supernatant after synthesis of the bionanocomposite prepared with 5 mM MgCl_2_ and before re- suspending the pellet in Tris-HCl pH = 7.5 and EDTA; 3) plasmid DNA desorbed from the bionanocomposite obtained with 5 mM MgCl_2_; 4) plasmid DNA desorbed from bionanocomposite obtained with 5 mM CaCl_2_.

Envisaging some possibility to transfer DNA into cells, it is important to verify that adsorbed DNA can actually be the released from the sepiolite nanofibers. Since DNA binds sepiolite through multivalent cations (see above), we aimed to desorb the DNA from the sepiolite by re-suspending the bionanocomposite in Tris-EDTA. Indeed, EDTA is a chelating agent, which retains the ability to chelate metal ions such as Mg^2+^, Ca^2+^. Here, we assumed that DNA desorption could occur through chelation of the cation bridges that bind DNA molecules to the sepiolite surface. We tested various concentrations of EDTA (Fig. 5A). Maximum DNA desorption was obtained with bionanocomposites Sep/DNA prepared in the presence of 5 mM MgCl_2_ and resuspended in 10 mM TrisHCl and 5 or10 mM EDTA. The efficiency of EDTA-mediated desorption was then compared with bionanocomposites synthesized with different cations, i.e. divalent, trivalent and tetravalent cations. Both, the total amount of DNA initially adsorbed and desorbed correlated with the valence of the cation present during the adsorption process (Fig. 5B). However, the percentage of DNA remained bounded to the sepiolite after desorption, clearly indicates that the desorption efficiency decreased with higher cations valence.

To estimate the quality of desorbed DNA, a Sep/DNA bionanocomposite was synthesized with a plasmid DNA (5.7 Kbp), in the presence of 5 mM Mg^2+^ or 5 mM Ca^2+^ ions. After desorption (10 mM EDTA), desorbed DNA was then analyzed by electrophoresis (Fig. 5C). Importantly, the different isoforms (super-coiled, linear, open circle) of the plasmid DNA, particularly the supercoiled form, were maintained in the DNA recovered from the sepiolite. The preservation of the DNA structure confirms that sepiolite could be a suitable support for the DNA, without altering its structure.

### Nucleic acids delivery into mammalian cells: proof of concept

First, we measured the capacity of sepiolite to deliver nucleic acids in human sarcoma cells. We took advantage of fluorescent siRNA to visualize the internalization of the nucleic acids into human cells. Second we analyzed the efficiency of stable plasmid delivery in two types of mammalian cells (hamster and human cells).

**Figure 6:**
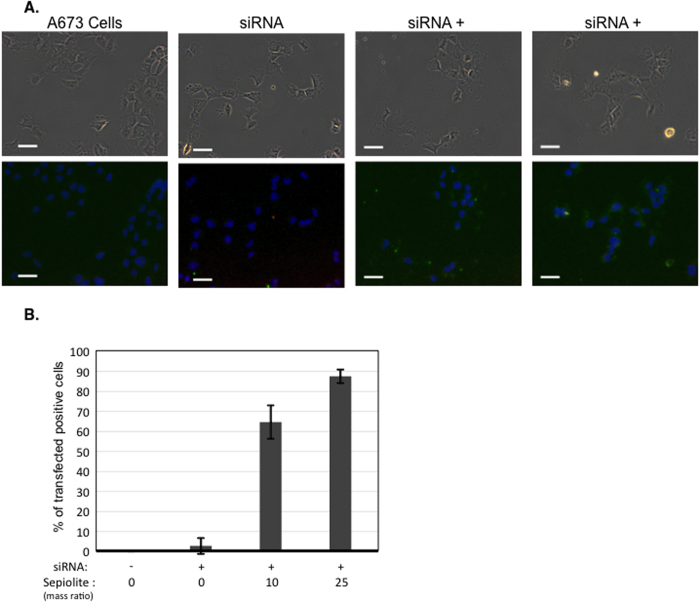
(**A**) Delivery of fluorescent siRNA by sepiolite in A673 human sarcoma cells. The cells are treated for 3 h alone (A673 cells) or with 50 nM FITC labeled siRNA (green) free or complexes to sepiolite at mass ratio of 10 or 25 (Sepiolite/siRNA; weight/weight). Cell nuclei are stained by DAPI (blue). Scale bar is 20 μm. (**B**) Efficiency of siRNA delivery by Sepiolite in A673 human sarcoma cells. After 3 h incubation, A673 sarcoma cells are fixed and observed by epifluorescence microscopy and the percentage of cell containing green fluorescent siRNA is determined on different slide fields for each one conditions of treatment.

To evaluate the efficiency of sepiolite for siRNA delivery into cells, we used a FITC labeled siRNA bounded to different mass ratio of sepiolite. Green fluorescence was observed neither in untreated cells nor in free siRNA treated cells (Fig. 6A). Interestingly, the association of siRNAs with sepiolite facilitated highly efficient transfer into cells, up to 90%, in an apparent dose dependent manner (Fig. 6A).

Since sepiolite adsorbs and desorbs DNA, it might constitute a carrier for DNA delivery into mammalian cells. In order to establish a proof of concept of this hypothesis, Sep/DNA bionanocomposites were prepared in the presence of 10 mM MgCl_2_ or CaCl_2_ using the plasmid pCMV, which encodes the gene for resistance to the G418 mammalian antibiotic.

**Figure 7:**
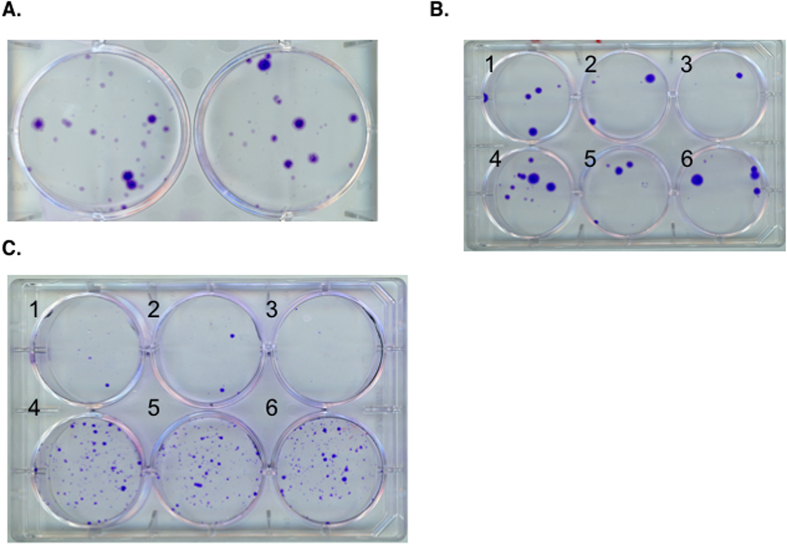
(**A**) Sepiolite-mediated stable gene transfer into mammalian cells. An image of colonies of transfected V79 cells with Sep/DNA prepared with 10 mM MgCl_2_ (left) and 10 mM CaCl_2_ (right). (**B**) An image of colonies of transfected V79 cells with Sep/DNA prepared with 10 mM CaCl_2_ (1, 2), 0.2 mM spermidine (3, 4) and 0.2 mM spermine (5, 6). First day: 2·10^4^ cells were plated in each well. Second day: 3 ml of new medium containing Sep/DNA bionanocomposite with 40 μg of sepiolite and 1 μg of bound DNA in the presence of polyvalent cation was added. After two days, the cells were washed with PBS and new medium was added with antibiotic G418 at 600 ng·μl^−1^ to start the selection. After 10 days, the cell colonies were stained with Giemsa (25% in ethanol). (**C**) A comparison of the number of colonies of transfected U2OS human cells with Sep/DNA (with vortexed sep in 1, 2 and 3), and sSep/DNA (with sonicated sep in 4, 5 and 6). First day: 10^4^ cells were plated in each well. Second day: 9 ml of new medium was added (3 ml in each well) in the presence of Sep/DNA (1–3) and sSep/DNA (4–6) bionanocomposites prepared from 80 μg of sepiolite and 3 μg of bound DNA in the presence of CaCl_2_. After two days, the cells were washed with PBS and new medium was added with antibiotic G418 at 800 ng·μl^−1^ to start the selection. After 10 days, the cell colonies were stained with Giemsa.

The DNA transfer efficiency of the Sep/DNA bionanocomposites into mammalian cells was further determined. Transfected cells were selected based on the acquired resistance to G418 as a result of the transferred plasmid bound to the sepiolite, following spontaneous uptake of the Sep/DNA bionanocomposite into cells. After 10 days of selection with G418, stable resistant colonies were obtained (Fig. 7A). These data show that the Sep/DNA bionanocomposite is actually able to transfer the plasmid DNA bearing G418-resistant genes into cells and can produce stable transfectants after several days of selection.

The transfection efficiencies of Sep/DNA bionanocomposites prepared in the presence of different cations were also compared. Thus, the presence of calcium chloride in the reaction medium results in bionanocomposites that slightly stimulated the efficiency of transfection compared to either spermidine or spermine (Fig. 7B), likely due to the strong interaction between DNA and sepiolite, which should impair the release of DNA from the bionanocomposites prepared in the presence of the polyamine cations. These experiments were performed in hamster cell line V79. We then tested another type of cell line, human osteosarcoma cells (U2OS). Interestingly, sepiolite was also able to transfer the plasmid DNA into another cell line (U2OS) of human origin (Fig. 7C). These data suggest that sepiolite-mediated DNA transfer should be generalizable to other mammalian cells (including human cells).

### Improvement of the transfection efficiency in mammalian cells

Sepiolite spontaneously aggregates. This aggregation may constitute a limitation for efficient widespread transfection applications. Therefore, the use of sonicated sepiolite (sSep). Indeed, sonication implies better disaggregation of sepiolite fibers than vortex alone. Therefore, this should increase the efficiency of interaction with DNA and cells. A series of bionanocomposites was synthesized with the plasmid and sonicated sepiolite (sSep/DNA). Sonicated sepiolite assembled with DNA results in bionanocomposites that show a strong increase (by two orders of magnitude) in the efficiency of stable gene transfection (Fig. 7C). Indeed, the mean transfection efficiencies were 3 and 350 stable transfectant colonies per μg of DNA, with non-sonicated and sonicated sepiolite, respectively. This result underlines the interest in the use of sonicated sepiolite, which results in systems that can show 100-fold higher efficiencies for stable transfection in mammalian cells.

### Conclusions and perspectives

Sep/DNA bionanocomposites were successfully prepared by the direct assembly of DNA and sepiolite and were characterized using a combination of techniques, including AFM, TEM, UV-visible spectroscopy, zeta-potential and FTIR. Notably, the DNA adsorption efficiency can be modulated by supplying polyvalent cations in the reaction medium. Interestingly, it has been also shown that sepiolite not only adsorb but also can desorb DNA, which constitute the two pre-requisite to use it as DNA carrier into cells.

Moreover, it is possible to design strategies for increasing the efficiency of DNA delivery. Therefore, because of its characteristics, sepiolite might represent a promising nano-carrier for DNA assembly and transfer into mammalian cells. Moreover, because sepiolite can bind both DNA and proteins, it might constitute an advantageous carrier (nanoplatform) for the co-delivery of these two types of biological molecules.

Because of the low cost of sepiolite, the easy and convenient synthesis methods designed in the present work, the possibilities of improving the transfection efficiency and additional promising developments, sepiolite should thus represent an attractive nanoplatform for DNA delivery into mammalian cells. Therefore, after the characterization of sepiolite-DNA interactions and the proof of concept of sepiolite-mediated DNA transfer into mammalian cells (with increased DNA transfer efficiency) shown here, the capacity of co-delivery with other biological molecules represent exciting challenges for future prospects.

## Methods

### Sepiolite

Pangel S9 consisting of a commercial sample of very high sepiolite content (>95%), provided with a cation exchange capacity value near 15 mequiv/100 g was obtained from TOLSA (Madrid, Spain). A sepiolite suspension of 2 mg·ml^−1^ was prepared in 10 mM Tris-HCl buffer, pH = 7.5, under vigorous vortexing at maximum speed for a minimum of 10 min to properly disperse the clay. Another 2 mg·ml^−1^ stock solution was prepared using a sonicated sepiolite (sSep) obtained by sonication of the sepiolite suspension, 3 times at 30% amplitude for 10 s each time using a Vibra-Cell 75042 from Bioblock Scientific, followed by autoclaving for the intracellular experiments.

### DNA

Different DNA molecules were used: a) a commercial preparation of low molecular weight DNA, from salmon sperm, was supplied by Sigma-Aldrich (31149–50G-F, lot# 0001393538); the lyophilized DNA was dissolved in 10 mM Tris-HCl, pH = 7.5, to produce a 1.9 mg·ml^−1^ solution. b) A circular DNA plasmid (5.7 kb, pCMV) was obtained by the amplification of a bacterial culture and was purified using the commercial kit from Macherey-Nagel; DNA PUC19 plasmid was supplied by New England BioLabs at 1 mg ml^−1^ (pUC19 Vector #N3041S, lot# 0361204. c) Single-stranded DNA custom oligos 15 nt long (ss-Oligo) were provided by Eurogentec (M13 sense 5′-gtaaaacgacggcca-3′ and M13 antisense 5′-tggccgtcgttttac-3′); the lyophilized solids were dissolved in MilliQ water to provide respective solutions of each single-stranded DNA sense. d) Double-stranded DNA oligos 15 bp long (ds-Oligo) were produced using an annealing reaction in the presence of 10X NE Buffer provided by New England Biolabs and heating for 3 min at 95 °C, following by a slow decrease in temperature, after which the buffer was changed to Tris-HCl 10 mM pH = 7.5 using the Micro Bio-Spin^TM^ chromatography columns.

### Synthesis procedure and sepiolite – DNA interactions

#### Study of the influence of cations on DNA adsorption onto sepiolite

The adsorption of DNA molecules onto sepiolite was first studied in experiments of adsorption under isothermal conditions at 25 °C. A set of eight aliquots of 50 μl of Sep/DNA mixtures was prepared by mixing 25 μl of the stock solution of sepiolite suspension (2 mg·ml^−1^), 5 μl of 10 mM Tris-HCl for the case of Sep/DNA alone, and 5 μl of 10 times concentrated solutions of 5 mM MgCl_2_, or 5 mM CaCl_2_, or 1 mM spermidine (S2501-5G, Sigma) or 1 mM spermine (118F-008215, Sigma) for the respective cases, and 20 μl of eight different concentrations of salmon sperm DNA solution. The final sepiolite concentration was set at 1 mg·ml^−1^, and the final DNA concentration ranged from 70 to 700 ng·μl^−1^. Then, the Sep/DNA mixtures were stirred for 24 h at 25 °C at 700 rpm using a Thermomixer (Eppendorf) and then centrifuged for 5 min at 5,000 rpm. Finally, the DNA concentrations in the supernatants were measured using a NanoDrop ND1000 spectrophotometer (UV-vis spectrometry). The experiments were carried out 3 times for each condition.

#### Adsorption of DNA molecules of different conformations

Several adsorption isotherms were determined using commercial salmon sperm DNA, plasmids, 15 nt ssOligo, and 15 bp dsOligo nucleotides (controlled by gel electrophoresis and optical density at 260 nm). The reaction conditions were fixed to 10 mM Tris-HCl, pH = 7.5, and 5 mM MgCl_2_. For each set of 3 experiments, a set of eight aliquots of 50 μl of Sep/DNA mixtures was prepared by mixing 25 μl of the 2 mg/ml of the sepiolite suspension, 5 μl of 50 mM MgCl_2_ and 20 μl of eight different concentrations of DNA solutions (between 100 and 1,600 ng·μl^−1^). All of the Sep/DNA mixtures were stirred for 24 h at 25 °C and 700 rpm using an Eppendorf Thermomixer. Finally, the mixtures were centrifuged for 5 min at 5,000 rpm, and the DNA concentrations in the supernatants were measured using a NanoDrop ND1000 spectrophotometer.

#### FTIR characterization

FTIR spectra were recorded with a Bruker IFS 66v/S FTIR spectrophotometer. Samples of 10 ml of pure sepiolite and Sep/DNA (a powdered material collected after centrifugation and drying at room temperature) were prepared as self-supporting films avoiding the use of additives such as KBr and then were placed in the sample holder and scanned from 4000 to 250 cm^−1^ with 2 cm^−1^ resolution. A set of samples was prepared in 10 mM Tris-HCl pH = 7.5, 1 mg/ml sepiolite and in presence of 1 mM spermine, 1 mM spermidine, 10 MgCl_2_, 10 mM CaCl_2_, 300 mM KCl or 300 mM NaCl. A second set of samples was prepared similarly but incorporating DNA (commercial salmon sperm DNA, Sigma) with a mean final concentration of 650 ng·μl^−1^, followed by stirring overnight at 25 °C and 700 rpm using an Eppendorf Thermomixer. Finally, the mixtures were centrifuged for 5 min at 5,000 rpm, the supernatants were removed, and the pellets were dried at room temperature.

#### Zeta-potential measurements

A set of 1 ml samples were prepared in 10 mM Tris-HCl pH = 7.5, sepiolite 1 mg/ml and in presence of 10 mM MgCl_2_, 10 mM CaCl_2_, 1 mM spermidine and 1 mM spermine. A second set of samples was prepared similarly but incorporating DNA (commercial salmon sperm DNA), with a mean final concentration 710 ng·μl^−1^. Zeta-potential measurements of 1 ml suspensions were performed on a Malvern Zetasizer Nano ZS. Z-average values in intensity at pH = 7 were used as the mean hydrodynamic size (Dh), and the zeta-potential was measured in a 0.01 M KNO_3_ solution. Either HNO_3_ or KOH was added to the solution to modify the pH.

### Cell culture

Chinese hamster cells were grown in dishes as monolayers in modified Eagle’s medium (MEM) containing 10% fetal bovine serum (FBS). U2OS human osteosarcoma cells were grown in dishes as monolayers in Dulbecco’s modified Eagle’s medium (DMEM) from Life Technologies containing 10% (v/v) FBS. Cells were incubated at 37 °C with 5% CO_2_ in air and 100% humidification. MEM, DMEM and FBS were purchased from Life Technologies^TM^.

### TEM imaging study

#### Sep/DNA imaging

Samples were prepared from 5 μL of sepiolite or Sep/DNA solution deposited for 1 min on a 600-mesh copper grid covered with a thin carbon film activated by glow-discharge in the presence of pentylamine^43^. Grids were washed with aqueous uranyl acetate 2% (w/v), dried with ashless filter paper and observed in dark-field mode with a tilted illumination, using a Zeiss 912AB transmission electron microscope. Images were recorded at magnifications of 50,000×, 85,000× and 140,000× with a ProScan 1024 HSC digital camera and iTEM acquisition software (Olympus Soft Imaging Solution).

### Cell transfection

#### Delivery of siRNA

Sarcoma cells A673 were seeded one day before treatment in DMEM medium (Life technology) containing 10% fetal calf serum and 1% penicillin/streptomycin (Life technology) in a 12 wells plate containing a coverglass. The cells were incubated at 37 °C in a moist atmosphere containing 5% CO_2_. For cells treatment, we used a 50 nM 3′-FITC labeled siRNA (Eurogentec) in HNC buffer (10 mM Hepes buffer pH 7.2, 100 mM NaCl and 10 mM CaCl_2_). The complexes siRNA sepiolite were formed by mixing equal volume of siRNA solution and sepiolite (mass ratio of 10 or 25; sepiolite/siRNA; weight/weight) in HNC buffer to obtained a 10x concentrated solution corresponding to the desired dose after dilution in Opti-MEM medium (Life technology). Then 500 μL of fresh medium or medium containing siRNA free or complexes to sepiolite were added to cells for 3 h incubation at 37 °C, 5% CO_2_ in moist atmosphere. The cells were then washed with PBS, and 1 mL of 4% formal solution in PBS was added for 20 min at room temperature. The cells were washed three times with PBS and mounted on slide with DAPI fluoromount G (SouthernBiotech) before being observed with an epifluorescence microscope (Observer; Zeiss). Untreated cells present only a blue fluorescence due to DAPI nucleus coloration. A673 cells were a generous gift from Dr. Elizabeth R. Lawlor (University of Michigan, USA).

#### Transfert of plasmid DNA

Sep/DNA bionanocomposite with 80 μg of sepiolite and 2 μg of bounded DNA was prepared in 70 μl of solution in the presence of CaCl_2_ in the order of 40 μl of sepiolite at 2 mg/ml, 14 μl of 50 mM CaCl_2_, 7.8 μl of 10 mM Tris-HCl and 8.2 μl of pCMV encoding for the resistance gene to G418 at 490 ng·μl^−1^. For the comparison of the efficiencies of bionanocomposites prepared in the presence of spermidine and spermine, the procedure was the same, except that 14 μl of 1 mM spermidine and 14 μl of 1 mM spermine were used, respectively. After 2 h of incubation at 25 °C and stirring at 700 rpm in an Eppendorf Thermomixer, 5.83 ml of cellular medium (MEM) was added and gently homogenized. Finally, 6 ml of bionanocomposite suspended in cellular medium was added to two wells (3 ml in each well) with 2.10^4^ of V79 cells in each well. After two days, the cells were washed with PBS and new medium was added with antibiotic G418 at 600 ng·μl^−1^ to start the selection process. After 10 days, cell colonies were stained with Giemsa (25% in ethanol). Hamster V79 cells were a generous gift of Proj. John Thacker (Medical Research Council, Radiation & Genome Stability Unit, Harwell, Oxfordshire OX11 0RD, England).

For human cells, a Sep/DNA bionanocomposite with 80 μg of sepiolite and 3 μg of bounded DNA was prepared in 70 μl solution by mixing 40 μl of sepiolite at 2 mg·ml^−1^, 14 μl of 50 mM CaCl_2_ and 16 μl of plasmid (pCMV) at 490 ng·μl^−1^. After 2 h of incubation at 25 °C and stirring at 700 rpm in a Thermomixer (Eppendorf), 8.9 ml of cellular medium (DMEM) was added and gently homogenized. Finally, 9 ml of bionanocomposite suspended in cellular medium was added to three wells (3 ml in each well) with 10^4^ of U2OS cells in each well. The experiment was performed with both vortexed and sonicated sepiolite. After two days, cells were washed with PBS and new medium was added with antibiotic G418 at 800 ng·μl^−1^ to start the selection. After 10 days, cell colonies were stained with Giemsa. Cell transfection was carried out in 6-well plates. U2OS cells were a generous gift of Dr. François Boussin (Institut de Radiobiologie cellulaire et Moléculaire, CEA, Fontenay-aux-Roses, Frances).

## Additional Information

**How to cite this article**: Castro-Smirnov, F. A. *et al.* Physical interactions between DNA and sepiolite nanofibers, and potential application for DNA transfer into mammalian cells. *Sci. Rep.* **6**, 36341; doi: 10.1038/srep36341 (2016).

**Publisher’s note:** Springer Nature remains neutral with regard to jurisdictional claims in published maps and institutional affiliations.

## Supplementary Material

Supplementary Information

## Figures and Tables

**Table 1:** The zeta-potential of suspensions prepared from sepiolite in the presence of polycations with and without DNA (from salmon sperm) in the medium.

Sample composition	Zeta-Potential (mV)	Bionanocomposite	Z-Potential (mV)
Sepiolite 1 mg·ml^−1^ 10 mM Tris-HCl pH = 7.5	−11.7	Sepiolite 1 mg·ml^−1^ DNA 710 ng·μl^−1^ 10 mM Tris-HCl pH = 7.5	−26.5
Sepiolite 1 mg·ml^−1^ 10 mM Tris-HCl pH = 7.5 10 mM MgCl_2_	−4.33	Sepiolite 1 mg·ml^−1^ DNA 710 ng·μl^−1^ 10 mM Tris-HCl pH = 7.5 10 mM MgCl_2_	−9.48
Sepiolite 1 mg·ml^−1^ 10 mM Tris-HCl pH = 7.5 10 mM CaCl_2_	−5.08	Sepiolite 1 mg·ml^−1^ DNA 710 ng·μl^−1^ 10 mM Tris-HCl pH = 7.5 10 mM CaCl_2_	−10.0
Sepiolite 1 mg·ml^−1^ 10 mM Tris-HCl pH = 7.5 1 mM spermidine	+6.28	Sepiolite 1 mg·ml^−1^ DNA 710 ng·μl^−1^ 10 mM Tris-HCl pH = 7.5 1 mM spermidine	−12.5
Sepiolite 1 mg·ml^−1^ 10 mM Tris-HCl pH = 7.5 1 mM spermine	+12.8	Sepiolite 1 mg·ml^−1^ DNA 710 ng·μl^−1^ 10 mM Tris-HCl pH = 7.5 1 mM spermine	−8.09
