# Supplementary material for: Physical interactions between DNA and sepiolite nanofibers, and potential application for DNA transfer into mammalian cells
Source: Sci Rep. 2016 Nov 3;6:36341. doi: 10.1038/srep36341 (PMC5093858; doi:10.1038/srep36341)
Supplement: Supplementary Information [file srep36341-s1.pdf]

Supplementary data

Physical interactions between DNA and sepiolite nanofibers, and potential application for DNA transfer into mammalian cells.

Fidel Antonio Castro-Smirnov, Olivier Piétrement, Pilar Aranda, Jean-Rémi Bertrand, Jeanne Ayache, Eric Le Cam, Eduardo Ruiz-Hitzky, and Bernard S. Lopez.

SUPPLEMENTARY DATA

Supplementary data S1

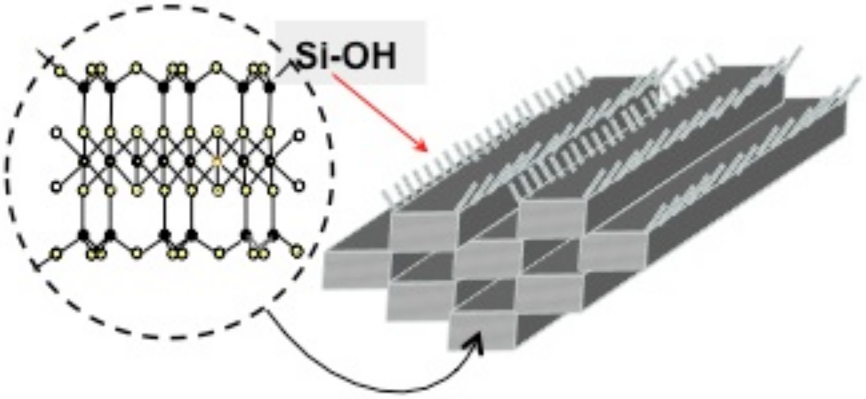

Figure S1. Scheme illustrating the cross-section of an elemental fiber of sepiolite showing the alternation of magnesium silicate blocks that defines the tunnels and channels of the structure and the location of silanol groups at the external surface.

Supplementary data S2

Time (min)	0	30	60	90	120	180	1440
µg of DNA adsorbed by 50 µg of Sepiolite	5.63	5.70	5.46	5.90	5.91	6.39	6.74
Percentage of adsorbed DNA	16.79%	16.99%	16.28%	17.59%	17.65%	19.07%	20.10%

Table S2. Time kinetics for DNA adsorption onto sepiolite (one experiment). Reaction conditions: 10mM TrisHCl PH=7.5, 5mM $MgCl_2$, sepiolite 1mg/ml, DNA 670.7 ng.μl^{-1}.

Supplementary data S3

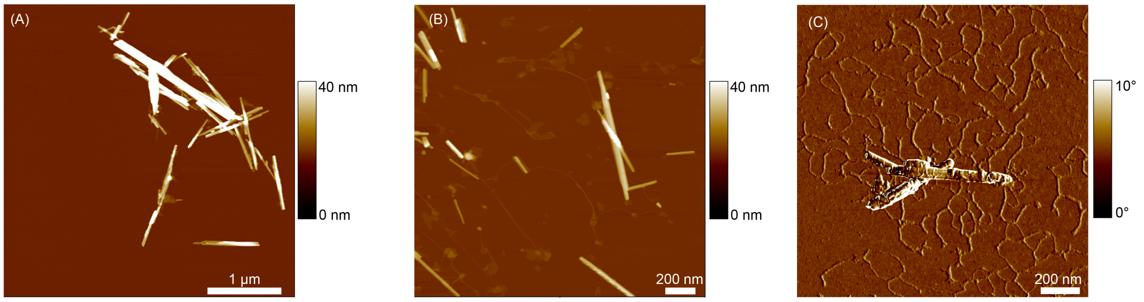

Figure S2. AFM images of sepiolite alone (A) and sepiolite/DNA (B) and (C, in phase mode).

Materials and methods

Atomic force microscopy (AFM) imaging of DNA/sepiolite assemblies was performed on a freshly cleaved mica surface treated with 50 µM spermidine for 1 min (1). Excess of spermidine solution was blotted with filter paper and 3-5 µL of 5 nM DNA sample was deposited on the mica surface, incubated for 1-2 min, and rinsed with 25 µL of 0.2 % (w/v) uranyl acetate[1]. The surface was blotted and dried. Imaging was carried out in Tapping Mode, with a Multimode system (Bruker) operating with a Nanoscope V controller (Bruker) using silicon AC160TS cantilevers (Olympus) with resonance frequencies of ~150 kHz. All images were collected at a scan frequency of 1 Hz and a resolution of 1024 × 1024 pixels. Images were analysed with Nanoscope V software. A third-order polynomial function was used to remove the background.

Reference :

(1) Hamon, L.; Pastré, D.; Dupaigne, P.; Le Breton, C.; Le Cam, E.; Piétrement, O. High-Resolution AFM Imaging of Single-Stranded DNA-Binding (SSB) protein–DNA Complexes. *Nucl Acids Res* **2007**, *35*, e58.
